# The B-side of *Xist*

**DOI:** 10.12688/f1000research.21362.1

**Published:** 2020-01-28

**Authors:** Asun Monfort, Anton Wutz

**Affiliations:** 1 Institute of Molecular Health Sciences, Swiss Federal Institute of Technology, Hönggerberg, HPL E12, Otto-Stern-Weg 7, Zurich, Switzerland

**Keywords:** Dosage compensation; chromatin; X inactivation; Xist; non-coding RNA; SPEN; Polycomb; gene repression

## Abstract

Female mammals express the long noncoding **X inactivation-specific transcript ( *Xist*) RNA to initiate X chromosome inactivation (XCI) that eventually results in the formation of the Barr body. *Xist *encompasses half a dozen repeated sequence stretches containing motifs for RNA-binding proteins that recruit effector complexes with functions for silencing genes and establishing a repressive chromatin configuration. Functional characterization of these effector proteins unveils the cooperation of a number of pathways to repress genes on the inactive X chromosome. Mechanistic insights can be extended to other noncoding RNAs with similar structure and open avenues for the design of new therapies to switch off gene expression. Here we review recent advances in the understanding of *Xist* and on this basis try to synthesize a model for the initiation of XCI.

## Introduction

The repression of one entire X chromosome in female cells achieves dosage compensation for X-linked genes between both sexes and has become a paradigm for studying epigenetic silencing. The mechanism by which hundreds of genes are repressed in a coordinated manner has captured the interest of researchers for over half a century but is by no means the only aspect of the mammalian dosage compensation system that remains a puzzling wonder of biology ^1^. The choice of the X chromosome to switch off, either maternal or paternal, is widely accepted to be random in placental mammals, and recent evidence in mice suggests a stochastic process for which a few components have been identified ^2, 3^. A mechanism for generating randomness is difficult to establish on a molecular basis but ultimately may inspire technology as well as cell biology. Recent progress in chromatin biology has, however, led to considerable insight into the pathways that accompany the silent fate of the inactive X chromosome (Xi). It is important to state from the outset that once the Xi has been chosen and repressed, its silent state is faithfully maintained and inherited in all progeny of every embryonic cell. This observation requires an epigenetic memory that is established at the Xi but has not been fully deciphered yet ^1, 4, 5^. The process of X chromosome inactivation (XCI) has been dissected into two characteristic phases: 1) a reversible X inactivation-specific transcript ( *Xist*)-dependent initiation step, when X linked genes become repressed, and 2) a maintenance step when gene silencing becomes irreversible and independent of *Xist* (reviewed in 6). Notwithstanding, a faulty initiation of the silencing process has consequences for the completion of the latter events, which demonstrates the exquisite functional coordination that is set in motion by *Xist*.

**Figure 1.**
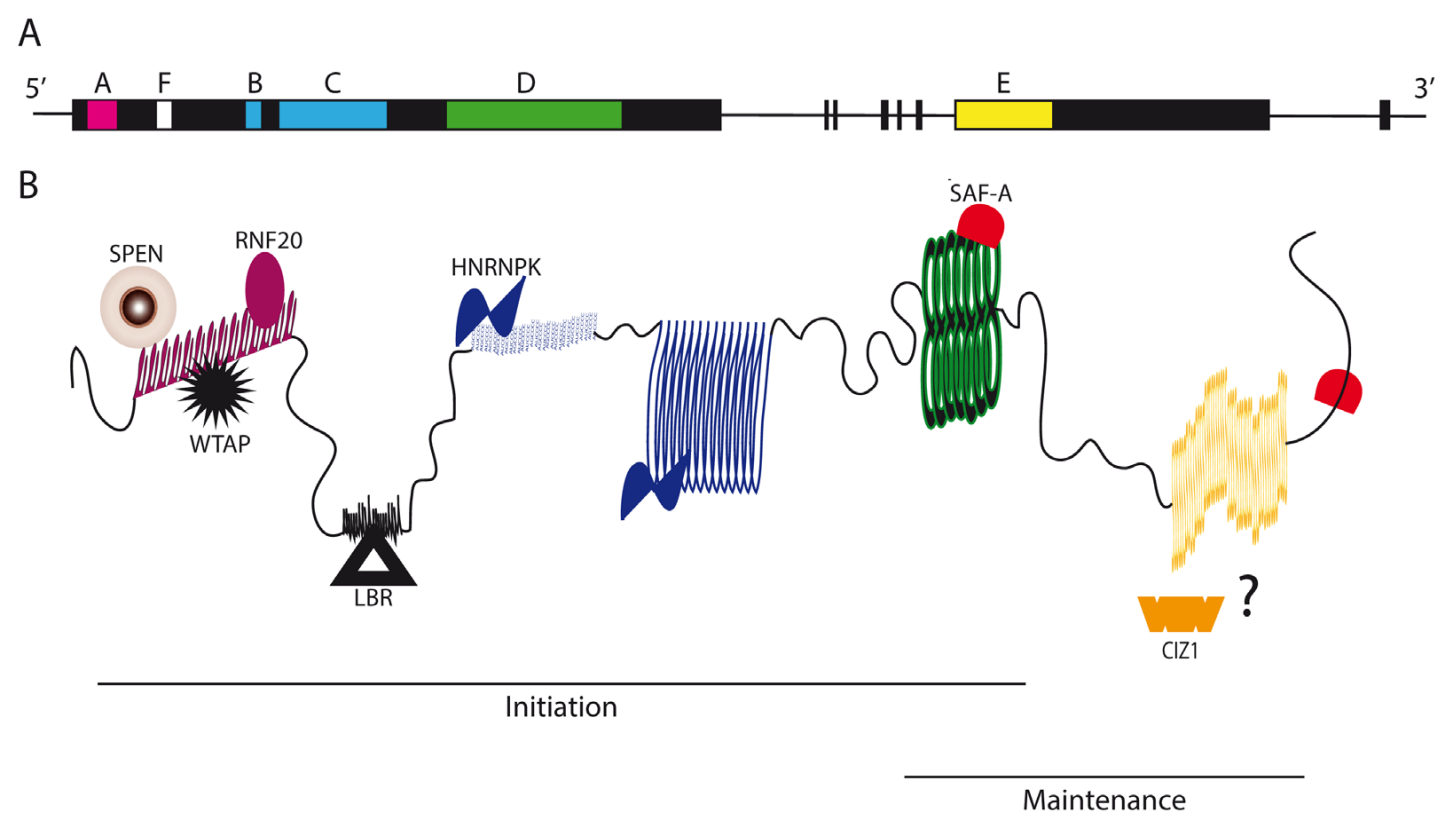
Repeat modules and interactors of *Xist* RNA. **A**) Representation of the mouse *Xist* gene. Exon1 codes for repeats A–D and F. Exon 7 codes for repeat E. **B**) Mouse *Xist* RNA with its functional A–F repeats and their corresponding direct interactors. Lines indicate the repeats predicted to contribute to the initiation and maintenance of XCI. The binding of CIZ1 to *Xist* has not been fully established and is indicated by a question mark. CIZ1, CDKN1A-interacting protein; HNRNPK, heterogeneous nuclear ribonucleoprotein K; LBR, lamin B receptor; SAF-A, scaffold attachment factor A; SPEN, Split Ends; RNF20, ring finger protein 20; WTAP, WT1-associated protein.

The 17 kb long noncoding RNA *Xist* is expressed from and accumulates over the X chromosome, triggering a cascade of events that eventually will result in the formation of the heterochromatic Barr body ^7– 11^. Within the *Xist* sequence, a number of repeat modules, named A to F, have been identified. These modules consist of unique multimeric short sequence stretches that play a functional role in the inactivation process ( Figure 1) ^7, 9, 12– 14^. Repeats A to D and F are encoded in *Xist* exon 1, whereas repeat E resides in exon 7 ( Figure 1A). In the last few years, a number of *Xist* partners have been identified, and their specific interaction with one of the repeat modules in *Xist* has been characterized ^6^. This has led to the establishment of a preliminary but highly interesting repeat-to-function correlation through which *Xist* is beginning to be better understood ^15^. Here, we review the latest advances in understanding mouse *Xist*, highlighting important insights and raising questions that remain unanswered.

## *Xist* repeat A is silencing

Close to the 5' end of *Xist* RNA, multiple repetitions of a short, conserved sequence are encountered that have been named repeat A ^9^. Each of the 24-nucleotide-long motifs contains small sequence inversions that possibly could fold into a double stem loop structure. The full mouse repeat A module would then comprise eight first-stem and seven second-stem loops ^13^. However, double-stem loop structures might not form within cells, and more complex conformations involving interactions between different 24-nucleotide-long core motifs have been proposed. Jones and Sattler provide an excellent review of the structure of *Xist* repeat A ^16^. Initial evidence for a role in gene silencing stems from a deletion analysis of mouse *Xist* using a transgenic system in embryonic stem cells (ESCs). These studies revealed the requirement of repeat A for the repression of X-linked genes but not for localization or spreading of *Xist* over the X chromosome ^13^.

Recently, the identities of some of the *Xist* partners responsible for repressing transcriptional activity were unveiled. Forward genetic screening and biochemical purification of *Xist* partners, in differentiated and undifferentiated cells, identified SPEN as a top candidate responsible for gene repression ^17– 20^. SPEN is a 400 kDa protein that belongs to the *Split Ends* family of transcriptional repressors. Its mutation in mice causes an embryonic lethal phenotype at E12.5 ^21^. SPEN contains four N-terminal RNA recognition motifs (RRMs) and a C-terminal conserved Spen paralog and ortholog domain (SPOC) that mediates the recruitment of co-repressors and the activation of histone deacetylases (HDACs). Different approaches have confirmed the direct interaction between SPEN and repeat A of *Xist* ^17, 19, 20, 22, 23^. Biochemical purification has shown that SPEN interacts with the full-length but not the repeat A mutant form of *Xist* ^19^. Deletion of exons encoding the RRM domains of SPEN abrogates the silencing capacity of *Xist* ^20^. In addition to a loss of gene silencing, *Xist* appears to lose the ability to recruit chromatin-modifying complexes of the Polycomb group ^17, 20^. A pathway for gene repression has been proposed based on the known interaction of SPEN with the SMRT co-repressor that activates HDAC3 ^17^. HDAC3-mutant ESCs show partial gene silencing upon *Xist* expression, suggesting that not all gene repression is mediated by HDAC3 ^24^. Taken together, these findings make SPEN a well-established silencing factor of *Xist*, though details of the mechanism in XCI remain to be uncovered.

Additional silencing factors for the initiation of XCI might be inferred from biochemical experiments that also indicate the interaction of RNF20 and WTAP with *Xist* in a repeat A-dependent manner ^19^. WTAP is a component of the RNA N-6 adenosine methylation (m ^6^A) complex that also contains RBM15, its paralogue RBM15B, and the methyltransferase METTL3 ^25^. RBM15 has been identified independently as an *Xist*-binding partner by genetic screening and biochemical purification ^17– 19^. Methylation individual nucleotide resolution crosslinking and immunoprecipitation (methylation-iCLIP) experiments in HEK293T cells revealed 78 m ^6^A residues in the human *XIST* RNA, some of which are localized in the vicinity of, and two of which are located within, repeat A ^26^. These sites also show prominent binding of the RNA m ^6^A reader YTHDC1. In ESCs, synthetic tethering of YTHDC1 to *Xist* was sufficient to complement the depletion of other WTAP complex members, which impairs gene repression by *Xist*. These findings indicate that m ^6^A acts as a recruitment signal for silencing factors ^26^. However, not all m ^6^A sites are localized at repeat A. In addition, a recent study has investigated the deletion of most m ^6^A sites at the 5' end of *Xist* and observed only subtle or no effects on X chromosome-wide gene silencing in mouse ESCs ^27^. This suggests that m ^6^A sites in the remaining *Xist* sequence may act redundantly for the recruitment of additional silencing factors. In mice, mutations in WTAP, RBM15, and YTHDC1 result in embryonic lethality ^28– 30^. Although these observations are consistent with a potential role in XCI, female-specific phenotypic dimorphisms have not yet been described. Further evidence would be desirable to establish the precise role of the WTAP complex at the initiation of X inactivation.

RNF20 is a third factor that has been identified to associate with *Xist* RNA in a repeat A-dependent manner ^19^. The RNF20/RNF40 family of E3 ubiquitin ligases can mediate mono-ubiquitylation of histone H2B lysine 120 (H2BK120ub1), which is required for mouse preimplantation development and ESC differentiation ^31– 33^. H2BK120ub1 has been correlated with both transcriptional activation ^34^ and repression ^35^ and is proposed to play a role in stabilizing nucleosomes and chromatin compaction ^36, 37^. Depletion of either RNF20 or RNF40 did not impair *Xist-*mediated silencing in mouse ESCs ^19^. Although a combined disruption of both RNF proteins has not yet been reported, it is tempting to speculate that RNF20 could contribute to chromatin modification and compaction in XCI.

### Repression independent of *Xist* repeat A

Most studies in mouse ESCs have confirmed a key role of repeat A in gene repression. However, studies of imprinted XCI in extraembryonic cells have uncovered the finding that repression of several genes on the Xi is apparently independent of repeat A. Forced expression of a repeat A mutant *Xist* from a heterologous promoter in trophoblast cells caused repression of genes that are not inactivated by the *Xist* repeat A mutant in embryonic cells ^38^. These repressed genes appear to cluster in regions of low gene density and are normally expressed at a low level on the active X chromosome (Xa). One potential explanation for the different requirement of repeat A in ESCs and trophoblast cells might come from considering the timing of *Xist* expression relative to gene activity: in the mouse embryo, *Xist* is first upregulated at the four-cell stage, concurring with the zygotic genome activation ^39^. At this stage, not all X-linked genes might be fully activated. It is conceivable that *Xist* without repeat A is able to repress some X-linked genes by chromatin modifications that normally contribute to the maintenance of gene repression in XCI. Therefore, the repeat A mutant *Xist* would not necessarily use an active silencing pathway but rather could rely on the maintenance pathways. Consistent with this idea is the finding that the expression of *Xist* containing repeat A has far greater potential for X-linked gene repression in trophoblast cells.

## The B-side of *Xist* brings in Polycomb

Mouse *Xist* repeat B consists of 32 copies of the cytidine-rich (A/U)GCCCC motif and functions together with repeat C, which contains 14 copies of a 120-nucleotide unit of high similarity ^40^. Recent studies have discovered key roles for *Xist* repeats B and C for the recruitment of Polycomb complexes.

### Polycomb repressive complexes: shedding light on the recruitment mechanism

*Xist* repeats B and C are required for the recruitment of canonical and non-canonical Polycomb repressive complex 1 (PRC1) and PRC2 to the Xi at the initiation of XCI. Work by the Brockdorff lab has unraveled a hierarchy for Polycomb complex recruitment by *Xist*. Initially, *Xist* repeats B and C recruit a non-canonical PRC1 that catalyzes the ubiquitination of histone H2A lysine 119. H2AK119ub is bound by RYBP, which is a component of a non-canonical PRC1 complex, and leads to an amplification and presumably spreading of the H2AK119ub mark. PRC2 activity is subsequently recruited and mediates H3K27me3. H3K27me3 in turn is recognized by CBX7, which is a component of canonical PRC1 complexes. Therefore, in XCI, PRC1 is recruited before PRC2 and multiple interactions amplify the spreading of both Polycomb complexes ^41^.

Initial evidence for a role of *Xist* repeat B in Polycomb recruitment was provided by the discovery of a function for JARID2 in XCI ^42^. Da Rocha and colleagues tested the recruitment of JARID2 in differentiating ESCs containing inducible *Xist* mutant transgenes with different deletions. *Xist* lacking repeats F, B, and C did not recruit JARID2, leading to a decrease in the recruitment of PRC2. Soon after that, JARID2 was identified as an essential component for PRC2 recruitment to PRC1-marked chromatin ^43^. Using autosomal *Xist-*inducible transgenes carrying a deletion of repeat B plus a partial deletion of repeat C, defined as the *Xist* RNA-Polycomb Interacting Domain (XR-PID), researchers reported a strong reduction in the accumulation of Polycomb complexes by *Xist* ^44^. Reassessment of these experiments in an endogenous inducible *Xist* system in XY ESCs showed that both repeat B and repeat C can efficiently recruit Polycomb proteins to the Xi and that a double deletion of repeats B and C leads to a complete abrogation of the accumulation of H2AK119ub and H3K27me3 at the Xi ^45^.

To understand this mechanism in detail, it was crucial to identify the non-canonical Polycomb group ring finger (PCGF) proteins PCGF3 and PCGF5, as they are required for the recruitment of Polycomb complexes by *Xist* ^46^. Fluorescence recovery after photobleaching (FRAP) experiments showed PCGF3/PCGF5 to form stable interactions with *Xist* RNA domains, and a double *Pcgf3/5* deletion resulted in the loss of *Xist*-dependent H2AK119ub1 and H3K27me3 deposition ^46^. Notably, *Pcgf3/5* double mutant mice present a female-specific lethality at E9.5, consistent with their requirement for dosage compensation ^46^. *Xist* repeats B and C have been linked to PCGF3/5–PRC1 through hnRNPK, which has been independently identified as an *Xist* interactor ^19, 47^ and shown to be required for Polycomb protein recruitment ^19^. hnRNPK is essential for early embryo development ^48^. It presents several KH RNA-binding domains that are predicted to mediate interaction with *Xist* repeats B and C and a K protein interactive region (KI) required to bind PCGF3/5–PRC1 ^49^. It has been shown that deletion of repeats B and C abolishes the interaction of *Xist* RNA with hnRNPK and secondarily with PCGF3/5–PRC1 ^45^. These experiments establish hnRNPK as the RNA-binding protein that links *Xist* to non-canonical PRC1, leading to a change of previous views from a direct binding of Polycomb proteins to RNAs ^50, 51^. PCGF3 and PCGF5 associate with hnRNPK and a catalytic RING protein for marking entry sites with H2AK119ub ^44^. The requirement of hnRNPK for early embryo development makes studies of its function difficult. However, synthetic tethering of hnRNPK, but not KI mutant hnRNPK, to transgenic *Xist* that lacks the XR-PID region restores the recruitment of H2AK119ub ^44^. In the future, it might be interesting to more specifically disrupt interactions between hnRNPK and *Xist* or PCGF3/5–PRC1 to understand if repeats B and C could have additional functions that have not yet been discovered. Taken together, these findings provide strong experimental support for a pathway of Polycomb recruitment by *Xist*. A modular architecture of *Xist* is also suggested, with repeats B and C forming a recruitment site for hnRNPK and Polycomb complexes. More research will be needed to understand to what extent Polycomb complexes contribute to gene repression and in particular if they function in maintaining gene silencing on the Xi.

### Darkness in the mechanism of spreading

Studies in ESCs indicate that *Xist* might initially localize in spatial proximity to its genomic locus in regions pre-marked with PcG decoration and low transcriptional activity ^52^. From these initial “entry sites”, *Xist* triggers the formation of a repressive compartment that is devoid of marks of active transcription including RNA polymerase II ^53^. These initial events are followed by the recruitment of Polycomb proteins to the Xi and a conformational change that subsequently permits spreading and silencing of gene clusters with high transcriptional activity ^52, 53^. Notably, the *Xist* mutant lacking repeat A is able to recruit Polycomb complexes to the Xi, suggesting that gene repression is not required for *Xist* localization and Polycomb recruitment ^4^. Recent evidence supports and extends this view, showing that repeat A mutant *Xist* localizes and mediates decoration over most of the Xi except for regions where active genes reside ^24^. Similar observations were made with HDAC3-mutant ESCs showing that spreading of Polycomb complexes is not observed over active gene loci *.* These results directly compare transcriptional activity with PcG recruitment and suggest that they are mutually exclusive. Zylicz *et al*. observed that deletion of repeat A causes a reduction in the deposition of H2AK119ub over the Xi. However, this effect was not recapitulated in HDAC3-mutant ESCs where PRC1 recruitment was unperturbed ^24^. These findings might provide a stringent test for the proposed mechanism for Polycomb recruitment and potentially also suggest a contribution from *Xist* repeat A in Polycomb recruitment.

### SMCHD1: a player in Xi architecture

One of the downstream effects of the recruitment of Polycomb complexes by *Xist* is the recruitment of SMCHD1 to the Xi. SMCHD1 was initially identified in a genetic screen in mice as a key player in XCI. SMCHD1-null embryos present a female-specific lethality between E10.5 and E13.5, with derepression of genes and hypomethylation of CpG islands on the Xi ^54– 56^. The interaction between *Xist* and SMCHD1 was also described through proteomics screening in MEFs ^47^, although this is likely not direct, as it depends on *hnRNPK* ^57^. Recruitment of SMCHD1 is dependent on *Xist* expression ^57, 58^. *Xist* repeats B and C are required for recruiting SMCHD1 to the Xi in ESCs at the transition between the initiation and the stable maintenance phase of XCI ^57^. Studies in female mouse embryonic fibroblasts (MEFs) suggest that SMCHD1 recruitment by *Xist* is dependent on PRC1 and H2AK119ub ^57^. Notably, the mutation of *Smchd1* results in a stronger recruitment of H3K27me3 to the Xi ^59^. At present, it is not clear how SMCHD1 can be recruited by PRC1 but insulate from the effects of PRC2. Furthermore, additional roles for *Smchd1* have been proposed in remodeling the 3D architecture of the Xi ^58– 61^. SMCHD1 has been postulated to regulate the transient reorganization of the Xa chromatin towards a bipartite Xi structure ^58^. Studies in *Smchd1*-deficient MEFs have shown that the overall structure of the Xi is largely unaffected in the absence of SMCHD1. However, genes that are reactivated become reorganized in a manner that resembles more the Xa than the Xi. Importantly, the Polycomb dependence of SMCHD1 recruitment to the Xi would suggest a requirement of Polycomb complexes for the establishment of a memory for the maintenance of XCI. Taken together, these data demonstrate that *Xist* repeats B and C can engage different effectors and they do this through hnRNPK and a non-canonical PRC1.

## Repeat D directs *Xist* to the Xi

One important factor for the precise localization of *Xist* RNA to the Xi is scaffold attachment factor A ( *SAF-A*/ *Hnrnpu*) ^62^. SAF-A binding has been described at the repeat D of *Xist* ^63^. Repeat D contains multiple copies of a 290-nucleotide-long motif that can mediate interaction with SAF-A. However, repeat D might not be the only element that contributes to *Xist* localization. Earlier studies indicated that several regions of *Xist* act in parallel or redundantly to *Xist* RNA localization ^13^. However, it remains to be shown if repeat D is sufficient for *Xist* localization or if other interactions are also required ^22^.

## Repeat E keeps *Xist* in focus

*Xist* repeat E consists of 50 highly variable copies of 20–25-nucleotide-long units that have been implicated in *Xist* localization in differentiated cells and maintenance of XCI ^40^. Insight into repeat E function comes from the identification of CIZ1 as a protein that is recruited to the Xi in somatic cells. CIZ1 is a nuclear protein that progressively associates with the Xi in a *Xist*-dependent manner during female ESC differentiation. Although *Ciz1* mutant mice do not show developmental defects, a highly penetrant lymphoproliferative disorder has been reported in adult females ^64^. This hematopoietic phenotype is consistent with an earlier report of leukemia development in female mice, when *Xist* is conditionally mutated in the blood system ^65^. The blood cell diseases have further been linked with dosage compensation defects ^64, 65^, suggesting a special context for XCI in blood cells ^66, 67^. Systematic deletion experiments over different *Xist* regions in ESCs and MEFs unveil that CIZ1 recruitment depends on repeat E ^64, 68^. Deletion of *Xist* repeat E in ESCs does not impair *Xist* localization or the recruitment of Polycomb complexes ^64, 69^. However, in *Ciz1*-mutant female MEFs, *Xist* is delocalized and Polycomb complexes are not recruited to the Xi. These defects in somatic cells are accompanied by a partial de-repression of X-linked genes ^64, 68^. Importantly, delocalization of *Xist* has also been observed in hematopoietic cells from *Ciz1*-mutant mice.

A recent study has uncovered an independent function for repeat E in the recruitment of the ASH2L Trithorax group protein ^69^, but the function of ASH2L for gene repression on the Xi remains largely unclear. Recruitment of ASH2L depends on *Xist* localization but is independent of repeat A ^5^. Taken together, current evidence suggests that repeat E acts as a binding site for factors that contribute to XCI maintenance. The cell-type-specific effects of the *Ciz1* mutation are intriguing. It would be interesting to explore if a mutation of repeat E would resemble the *Ciz1* mutation in mice. Alternatively, other repeat E binding factors could be involved in either maintenance or transition to maintenance of XCI.

## *Xist* repeat F makes the lamina connection

*Xist* repeat F was not included in the initial description of *Xist* ^9^ but has been more recently defined as a short fragment that is located just 3' of repeat A. Repeat F is composed of two copies of a 10-nucleotide-long G/C-rich sequence motif (UGGCGGGCUU) ^40^ and appears to function together with a region near repeat E as a binding site for lamin B receptor (LBR) ^70^. LBR is a 60 kDa integral membrane protein embedded in the inner nuclear membrane that associates with lamin B and tethers heterochromatin to the nuclear periphery ^71^. Mutation of *Lbr* has been described to result in an embryonic lethal phenotype with incomplete penetrance, but a female-specific phenotype has yet to be described ^72, 73^. LBR was identified as a direct binder of *Xist* through a biochemical screening approach ^17^. RNA interference (RNAi)-mediated depletion of *Lbr* in ESCs was reported to cause an impairment of gene repression by *Xist*. These observations were further corroborated by showing that deletion of the LBR-binding sites of *Xist* resulted in a loss of gene repression ^70^. Tethering of LBR to *Xist* lacking the LBR interaction site could restore silencing activity. In addition, tethering a mutant form of LBR that lacked the RNA-binding domain to *Xist* complemented the silencing defect. Notably, LBR does not possess a canonical RNA-binding domain and associates with *Xist* through a novel surface. LBR might contribute to gene repression by mediating an association with the nuclear lamina. It is conceivable that also an association with a repressive heterochromatic environment at the nuclear lamina or reshaping of the 3D architecture of the Xi might play a role in XCI. However, a recent study has investigated a deletion of the *Lbr* gene in ESCs and observed only subtle silencing defects ^27^. In addition, after removal of the entire *Xist* repeat F, only minor defects in gene repression were observed. These observations are conflicting with earlier observations and suggest that additional evidence will be necessary to understand the requirement of *Lbr* for gene silencing. Nevertheless, repeat F might provide a binding site for *Xist* partners that engage additional mechanisms that are not yet fully understood.

## Conclusion

The understanding of chromosome-wide silencing on the molecular level has seen impressive advances over the past few years. Progress has been driven by the development of new methodology in RNA protein biochemistry and mammalian genetics. A large number of factors have been identified that cannot all be discussed in this review for space reasons. We focus here on factors that have been implicated and shown to be relevant for understanding *Xist* function. The current understanding suggests that *Xist* RNA might act as a link between different functional effector complexes. Engaging a number of molecular activities would then bring about the astonishing properties of *Xist*, namely its ability to specifically localize over the Xi and its ability to trigger chromosome-wide chromatin modifications and gene repression. This poses the following question: is the current knowledge of known *Xist* effectors sufficient for reconstitution of *Xist* function? The identification of the binding sites within *Xist* could make experimental testing possible as of today ( Figure 1B). Certainly, one would include some form of repeat A for engaging the gene silencing pathway. An important question here is whether tethering of SPEN would fully substitute for all functions of repeat A or whether WTAP and METTL3 would be required too.

For repeats B and C, the situation appears less clear. Would Polycomb engagement be required for initiating gene repression? There is evidence that XCI could initiate without Polycomb recruitment, and a repeat B and C mutant *Xist* can initiate gene repression ^45^. However, this view might change during the maintenance phase of XCI. Without repeats B and C, SMCHD1, and likely other factors, would not be recruited and establishing an epigenetic memory could potentially fail. This idea would suggest a role for Polycomb complexes in establishing a repressive memory in XCI, which is consistent with the traditional view of Polycomb function. Notably, interactions of the PRC2 subunits Ezh2 ^50^ and Suz12 ^51^ with *Xist* repeat A have previously been observed *in vitro*. However, the relevance of these observations for XCI remains unclear. Although Polycomb proteins might bind RNA directly, the requirement of hnRNPK in XCI suggests that these interactions are not sufficient in cells. Early studies have further proposed a role for repeats B and C in *Xist* localization. Jeon *et al*. implicated YY1 as a DNA- and RNA-binding protein that bridges *Xist* repeats B and C RNA to repeat F DNA, facilitating the localization of *Xist* ^74^. Consistent with this observation, peptide linked nucleic acids and locked nucleic acids complementary to repeat C sequences also induced delocalization of *Xist* ^75, 76^. These observations are puzzling when one considers that the deletion of repeats B and C does not abrogate *Xist* localization or spreading at the initiation of XCI ^13, 45^. To reconcile these conflicting interpretations, one might consider different cellular contexts or experimental conditions similarly to the cell-type-specific requirement of *Ciz1* for *Xist* localization ^64^.

Repeat D could be handy for engineering RNAs that mimic the localization of *Xist* ^77^. However, it remains to be shown if repeat D is sufficient for *Xist* localization or other interactions are also required such as with LBR. A prominent role of *Xist* repeat E in the maintenance but not in the initiation step of XCI has been uncovered through the identification of *Ciz1*. We speculate that both repeat D and repeat E could be important for the maintenance of XCI, where they might recruit factors independent of gene silencing but dependent on a chromosomal memory that is imposed by Polycomb complexes. Thus, repeats D and E of *Xist* could act as docking sequences for factors that preserve the integrity of heterochromatin of the Barr body and facilitate *Xist* localization in a cell-type-specific manner. Taken together, the current understanding of molecular interactions of *Xist* appears to provide a detailed guide for reconstitution of *Xist* function. Performing these experiments will be important for establishing if the main mechanistic components have indeed been identified or key factors are still missing. In the latter case, a number of unstudied proteins from the list of *Xist* interactors represent a resource for further exploration.

### *Xist* tunes the chromatin

**Figure 2.**
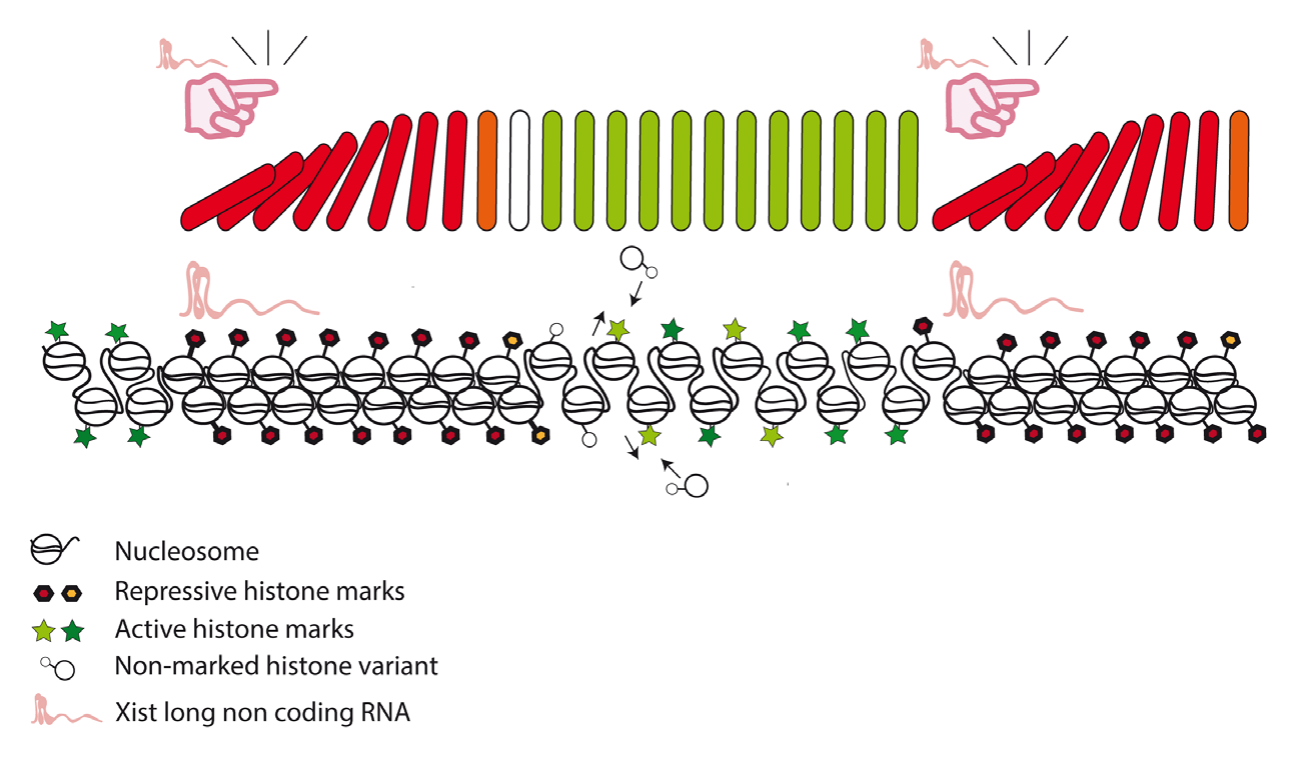
A proposed model for *Xist* triggering the heterochromatinization of a genomic fragment in a domino-like effect. *Xist* RNA induces local chromatin modifications that spread along the chromosome through an unidentified inter-nucleosomal communication mechanism.

Progress in understanding the molecular functions of *Xist* has moved the field towards a new frontier: the chromatin. Conceptually, *Xist* can be seen as a sender of a message. This view would predict a receiver on the chromosome. How X-linked gene promoters are inactivated on the chromatin level remains an open question. Just a few hundred molecules of *Xist* accumulate over the Xi and are capable of switching active genes and regulatory regions from an open into a closed chromatin configuration along an entire chromosome. This switch implies that the chromatin has the ability to read instructions from *Xist* and coordinate gene repression. Although some of these instructions come in the form of histone modifications, it remains unclear how silencing is spread. It is enticing to speculate if a domino-like effect might be triggered by *Xist* that perpetuates changes along the chromatin fiber. Would this make histones domino pieces? Histone exchange can facilitate switching an active into a repressive chromatin configuration through the removal of actively marked histones. Besides, gene silencing is accompanied by compaction of the chromatin fiber and prevents regulatory proteins from binding and triggering transcriptional events. We speculate that nucleosome assembly and chromatin dynamics might be important aspects for silencing and spreading of repressive chromatin modifications ( Figure 2). The factors that allow chromatin to respond to the messages of *Xist* remain to be discovered. Identification of these components can be predicted to have wide implications for the understanding of gene regulation in mammals.

The molecular pathways used by *Xist* for the generation of a silent and stable Barr body are starting to be elucidated, albeit much remains to be understood. Disrupting X-linked gene silencing leads to female embryonic lethality, showing the importance of this phenomenon for the survival of mammalian species. Functional characterization of the different effectors engaged by *Xist* can advance our understanding of the striking mechanisms that were selected by nature to warrant gene silencing for survival. This knowledge can be exploited therapeutically for the treatment of genetic disorders where a gene overdose impairs the correct function of cells, such as Down syndrome cell pathogenesis ^77^, X-linked diseases ^78^, or approaches involving cell reprogramming ^79^. Thus, further unraveling the mechanisms used by *Xist* and its effectors for silencing and heterochromatinization will contribute to advances in science and medicine.

## Abbreviations

ESC, embryonic stem cell; HDAC, histone deacetylation complex; MEF, mouse embryonic fibroblasts; PCG, Polycomb complex group; PRC, Polycomb repressive complex; RRM, RNA recognition motif; Xa, active X chromosome; XCI, X chromosome inactivation; Xi, inactive X chromosome; *Xist*, X inactivation-specific transcript.
